# Foreign Bodies in Pharynx and Larynx

**Published:** 1894-02

**Authors:** Arthur G. Hobbs, George Brown

**Affiliations:** Atlanta, Ga.; Atlanta, Ga.


ATLANTA
### MEDICAL AND SURGICAL JOURNAL.

VOL. X. FEBRUARY, 1894. No. 12.

EDITED BY
LUTHER B. GRANDY, M. D., AND MILLER B. HUTCHINS, M. D.,
WITH THE CO-OPERATION OF
H. V. M. MILLER, M. D., LL. D., VIRGIL O. HARDON, M. D.,
FLOYD W. McRAE, M. D., AND ARTHUR G. HOBBS, M. D.

### *ORIGINAL COMMUNICATIONS.*

### FOREIGN BODIES IN PHARYNX AND LARYNX.

### REPORT OF CASES.

BY ARTHUR G. HOBBS, M. D., AND GEORGE BROWN, M. D.,
ATLANTA, GA.

It has been truthfully said that a list of foreign bodies that have been in and removed from the pharynx and larynx would comprise most everything that the mind of man could think of. The varied substances that have been removed by different surgeons would comprise a rare and curious collection, ranging from steam engines (toy) to leeches, and from cedar pencils to pen knives.

We will instance a few cases from our note book, embracing some of the foreign bodies most frequently found in this region.

CASE No 1.

*Pin in the Pharynx.*—Six weeks after the accident, I detected what proved to be a pin at the bottom of a large abscess, in the lower pharynx, just below the right posterior pillar in a woman, aged thirty-two. The entrance of the foreign body was unknown to the patient until after its discovery and extraction. The persistence of the many aggravated symptoms and the abscess itself caused the patient to go through several hands before I saw her. Fortunately, in my efforts to probe the abscess to the bottom I found its cause and extracted the pin at the first sitting; healing rapidly followed.—[A. G. H.]

CASE NO. 2.

*Pin in Larynx.*—A girl, aged fourteen, accidentally got a pin lodged in her larynx. The pin pierced false band near its inner edge, thence across the glottic slit and through the right cord. The history and symptoms of this case were very similar to the case below of tack in larynx. After much difficulty, on the following morning, the pin was grasped midway with a McKenzie forceps and forcibly dislodged. The œdema rendered it impossible to see the head of the pin, but the consequent laceration produced but little bleeding, although the pin came out bent almost double. But little aphonia resulted and none of the dangerous symptoms of the case below followed the immediate extraction of the foreign body.—[A. G. H.]

CASE NO. 3.

*Tack in Larynx.*—John H., aged twenty-two, by a sudden inhalation, drew an ordinary carpet tack into the glottis. The sharp end penetrated the left cord and the head lodged beneath the right. When I first saw the patient, twenty-four hours after the accident, the œdema had become so great that asphyxia seemed imminent. This condition had been gradually increasing for twelve hours. Instruments were ready for a tracheotomy, and finally after many repeated attempts I obtained a glimpse of the condition of the glottis and the position of the foreign body, when by repeated trials, though not succeeding again in getting a good view, I grasped the tack about midway between its point and head with a modified duck bill forceps. Considerable force was necessary to tear it from its œdematous bed; an alarming paroxysm of coughing followed with considerable bleeding. Each minute at this time was an anxious one. Both O'Dwyer's tube and tracheotomy instruments were ready for immediate use, but after some moments the bleeding had so much reduced the glottic stenosis that the patient passed the dangerous point and slept after a few hours. Resolution was rapid, except that aphonia lasted about six weeks.—[A. G. H.]

CASE No. 4.

*A Cocklebur in Larynx.*—A boy, aged eight, was brought to my office by two physicians, that I might make a laryngotomy for the removal of a cocklebur, but after a cocaine spray and a laryngoscopic examination, the bur was seen to be lodged just beneath the glottic opening and was extracted without much difficulty with the long curved forceps. Strange enough, there was no aphonia, the voice only showing a slight hoarseness for a few days. Recovery was complete.—[A. G. H.]

CASE No. 5.

*Foreign Body in Nasal Passage.*—By a mere coincidence the next case I saw wasone of complete aphonia, in a child of four years, produced by the lodgment of a shoe button in the post-nasal space. The foreign body, from the history of the case, had been pushed into the nose four months previously by the little one and the loss of voice had existed for six weeks. The sequel proved that the foreign body was the cause of the aphonia as the first effort of vocalization after its extraction was successful. The voice continued good. The reason for mentioning the above case in this connection is obvious. There was no aphonia in the case, when the cords were directly involved, andyeta total loss of voice followed the distant lesion in this case. This is the so-called laryngitis, that is found so often (?) and exists so seldom; of the so-called laryngitis, perhaps ninety per cent. are due to nasal reflexes and exist only through subjective symptoms.—[A. G. H.]

CASE No. 6.

*A Piece of Meat in Glottis.*—Gentleman, aged sixty-six, farmer, eating breakfast of "spareribs," suddenly felt a choking sensation; living ten miles from the city, he was hurriedly driven into town for relief. As he had a very sensitive throat, I first sprayed it well with a cocaine solution, then found imbedded in the glottis what proved to be a very large piece of meat, very tough and fibrous. It was easily removed with long curved forceps, giving the patient instant relief and use of his voice.—[G. B.]

CASE NO. 7.

*A Piece of Bone in Larynx.*—A gentleman, aged thirty-five, while eating a luncheon composed of a beef stew, suddenly felt something slip into his larynx. After using a cocaine spray, a piece of bone was seen lying across the glottic slit, one end of which was imbedded in the ari-epiglottic fold between the base of the epiglottis and the cartilage of Wrisberg. The bone being perfectly white, was easily seen and as easily removed. It was triangular in shape, nearly three-fourths of an inch in length and tapered to a very sharp point at the apex; perfect recovery.—[G. B.]

We are often consulted by patients who think they have a foreign body in the throat and are loathe to believe that such is not the case. I will mention a few such instances:

CASE NO. 1.

A negro women, aged thirty-five, said she had a fish-scale in her throat that had been troubling her for fifteen years. Nothing whatever could be found, except a sensitive area of pharynx. Decided hypertrophy of the middle turbinate bone on the affected side, which was in my opinion the cause of her symptoms; she refused any treatment and I saw her no more.—[G. B.]

CASE NO. 2.

Negro waiter, aged forty, had swallowed a dime given him as a "tip" by a gentleman eight years ago. It was a very migratory coin as at times he felt it on one side and suddenly it would change to the other. When I saw him it was behind the palate, and in a few moments it was in the frontal sinus and shortly afterwards in the larynx. Of course no dime was found, much to his disgust. A general atrophic condition of the upper air passages was responsible for the different resting places of the coin.—[G. B.]

CASE NO. 3.

Mayor of adjoining town, aged thirty, was walking down the street and suddenly felt something drop into his larynx. He at once suffered extreme discomfort in breathing, in fact almost suffocated; he was badly frightened when I saw him a few moments later. The most thorough examination failed to discover any foreign body in the larynx, and as he had nothing in his mouth at the time, nor had their been since morning, I could only attribute it to a spasm of the glottis. A spray of camphor mentholine gave instant relief.—[G. B.]

Case No. 4.

Engineer, aged twenty-eight. While walking down the street in a snow-storm, the handle of his umbrella became loosened from the staff, and as it was attached with glue he put the staff in his mouth to moisten the glue in order to replace the handle. At that instant a gentleman ran against the other end of the umbrella and drove it with considerable force into his mouth. I saw him a few moments afterwards, and found that the end of the staff had passed completely through the soft palate and a small piece of flesh was hanging down, nearly on a level with the base of the tongue, this was clipped off with a pair scissors. The patient insisted that there was another piece in his larynx but I failed to find any foreign body there.—[G. B.]

